# Pain, depression and the postoperative stiff shoulder

**DOI:** 10.1186/s12891-015-0841-6

**Published:** 2015-12-04

**Authors:** Nathaniel Hiscock, Simon Bell, Jennifer Coghlan

**Affiliations:** Faculty of Medicine, Nursing and Health Sciences, Monash University, Melbourne, VIC 3800 Australia; Department of Surgery, Monash Medical Centre, Faculty of Medicine, Nursing and Health Sciences, Monash University, Melbourne, VIC 3800 Australia; Melbourne Shoulder and Elbow Centre, 31 Normanby St, Brighton, VIC 3186 Australia

**Keywords:** Postoperative stiff shoulder, Shoulder, Surgery, Subacromial decompression, Excision of distal clavicle, Distal clavicular resection, Rotator cuff repair, Pain, Depression

## Abstract

**Background:**

The surgical repair of shoulder pathologies, including rotator cuff disease and acromio-clavicular joint arthritis, have undergone many technical advances. However the debilitating postoperative stiff shoulder remains a common and significant complication of these surgeries, occurring in 4.9 to 23.2 % of patients undergoing rotator cuff repairs.

The pathology of the pathological postoperative stiff shoulder and its associated condition “frozen shoulder” are poorly understood and both lack formal objective clinical diagnostic criteria. Additionally, although factors associated with the development of idiopathic frozen shoulder have been well described, multiple studies looking at predictors of postoperative stiff shoulder have produced conflicting results. It has been hypothesised that increased pain in the postoperative period, and depression may be predictors of the development of postoperative stiff shoulder.

**Method:**

A prospective cohort study involving 132 consecutive participants. Preoperatively, participants undergoing arthroscopic subacromial decompression and/or excision of the distal clavicle and/or rotator cuff repair will complete questionnaires about their levels of shoulder pain using a numerical rating scale from 0 to 10, and answer a Patient Health Questionnaire – 9 depression questionnaire. Postoperatively, the participants’ pain levels will be self-assessed at two, five and seven days and weeks four, seven and ten. They will complete the depression questionnaire twice, at the time of their routine first and final postoperative appointments with the treating surgeon. At the final appointment, approximately three months postoperatively, the treating surgeon will clinically diagnose participants as having a postoperative stiff shoulder or not. Their shoulders’ range of motion will be measured. The incidence of postoperative stiff shoulder will be determined, both pain and depression will be analysed as predictors for its development and incidences determined by different objective criteria will be compared.

**Discussion:**

This trial will add to clinical understanding of the postoperative stiff shoulder by providing further insight into the incidence of this condition following shoulder surgery and assessing whether perioperative pain and depression can be used as clinical predictors of postoperative stiff shoulder or markers for possible early intervention. This study will also allow the comparison of incidences determined by different objective criteria in the same cohort.

**Trial registration:**

Australian New Zealand Clinical Trials Registry (ANZCTR).

ACTRN12613001271796.

17-11-2013.

**Electronic supplementary material:**

The online version of this article (doi:10.1186/s12891-015-0841-6) contains supplementary material, which is available to authorized users.

## Background

The enigmatic “frozen shoulder”, since its description by Duplay in 1872, has lacked a consensus definition until 2011 when Zuckerman established a consensus definition and classification system of frozen shoulder where he classified cases as either primary (idiopathic) or secondary [1–3]. He further delineated secondary frozen shoulder according to its associated aetiology, in which postoperative stiff shoulder (PSS), if viewed as a type of “frozen shoulder”, would fall under the intrinsic category. A survey of the members of the American Shoulder and Elbow Surgeons found that both this definition and classification were well received [3].

Zuckerman’s accepted definition of frozen shoulder is “a condition characterized by functional restriction of both active and passive shoulder motion for which radiographs of the glenohumeral joint are essentially unremarkable except for the possible presence of osteopenia or calcific tendonitis.” This descriptive definition is also an appropriate definition for postoperative stiff shoulder in the setting of patients who have recently undergone shoulder surgery.

Although the clinical features of frozen shoulder and PSS have been well characterised, a consensus of objective clinical diagnostic criteria has not been established [4]. A multitude of criteria has been published demonstrating a variety of methods used to diagnose patients with this condition. These include a marked variety of range of motion measurements with different thresholds [5–19] and clinical judgement with and without guiding criteria [11, 20–25] and contentiously patient satisfaction with outcomes [26]. The lack of universal, or even widely used, definitive criteria makes accurately comparing and synthesizing results from studies assessing frozen shoulder and/or PSS difficult to impossible and is possibly a reason behind reported rates of PSS varying between 4.9 and 23 % [5, 10, 13, 26]. Additionally in patients with suspected PSS it may be difficult to clinically distinguish pathological stiffness due to capsulitis from restricted motion due to a tight tendon repair and different criteria may over or under diagnose true pathology.

In our review of the literature surrounding frozen shoulder and PSS we found an array of criteria for both frozen shoulder and PSS. Objective criteria consisted of range of motion thresholds [5, 8, 10, 11, 13, 15, 18, 19, 27], global range of motion deficits compared to normal values [16], and range of motion deficits compared to the contralateral side [6–9, 11, 12, 14, 15, 17, 19]. We believe that the pursuit of a consensus objective criteria for PSS, and frozen shoulder, is important and we aim to apply various criteria to our patient cohort to determine their sensitivity and specificity of each test for the treating surgeon’s clinical diagnosis. We hope that these results will stimulate discussion on this issue.

We also reviewed the literature for studies researching the risk factors of PSS post rotator cuff surgery and found a number of studies, whose results conflicted [5, 10, 13, 22, 26, 28]. There is a scarcity of literature analysing the incidence of PSS after subacromial decompression or excision of the distal clavicle not in association with rotator cuff repair.

There are a number of potential risk factors for frozen shoulder and PSS that have been studied. These include sociodemographic, medical and psychological factors. Age, a traditional risk factor for primary frozen shoulder has been found to be and not be associated the development of PSS, with both higher and lower age associated with PSS in separate studies [10, 13, 28]. Our review found multiple studies associating worker’s compensation claims with the development of PSS, however one study rebutted these findings [22, 26, 28].

Diabetes, another traditional risk factor for primary frozen shoulder, has been found to be and not be associated with the development of PSS [10, 22]. Multiple studies have shown that patients with PSS after rotator cuff surgery have higher pain levels at 12 weeks with one study also finding that higher pain levels at six weeks were associated [10, 22, 28]. No studies have assessed the relationship between pain levels in the first month postoperatively, the time when the initial inflammatory healing process begins, and the development of PSS.

Anecdotally, psychological factors have long been believed to be associated with the development of frozen shoulder. Codman proposed a link between one’s constitution and the development of “frozen shoulder” when he first coined the term in 1934 and Coventry used the term “periarthritic personality” to describe these personal characteristics who “were a little run-down without anything particular the mater” [2, 29]. A number of authors have since looked at both personality traits and the mental health of patients with primary frozen shoulder [21, 25, 30–34]. The findings from these studies are conflicted and inconclusive and rely upon varying measures of personality and depression, although recent studies found that the mental health of patients with primary frozen shoulder was no different to that of the general population [32, 33]. No research has been done assessing a temporal relationship between depression and frozen shoulder’s related condition PSS. Given these historical views, depression’s known association with chronic pain and disability, and the described association of PSS with worker’s compensation claims we suggest that it is possible that patients who are depressed will be more likely to develop PSS [22, 26, 27].

The three aims of this study will be first to determine the incidence of postoperative stiff shoulder in patients of a single surgeon following arthroscopic subacromial decompression, and/or excision of the distal clavicle and/or rotator cuff repair rotator cuff repair; secondly, we will analyse the relationship between perioperative pain and depression, assessed by the Patient Health Questionnaire – 9, and the development of postoperative stiff shoulder, in patients following arthroscopic subacromial decompression, and/or excision of the distal clavicle and/or rotator cuff repair rotator cuff repair; thirdly, we will compare and contrast the incidences of postoperative stiff shoulder determined by different objective clinical criteria in the same patient cohort.

We hypothesize that patients who exhibit higher pain levels in the first month postoperatively will be more likely to develop PSS due to a more active postoperative inflammatory process. Secondly, we hypothesize that patients with depressed mood will be more likely to develop postoperative stiff shoulder. Thirdly, we hypothesize that different objective clinical criteria will determine different rates of postoperative stiff shoulder in the same patient cohort.

## Method

### Design

This will be a prospective cohort study observing patients who will undergo surgery including arthroscopic subacromial decompressions and/or excision of the distal clavicle and/or rotator cuff repair.

### Setting and participants

Over a six-month period participants will be recruited from the private community practice of a single orthopaedic shoulder surgeon (SNB) in Melbourne, Australia. Recruiting patients from a single shoulder surgeon eliminates the potential confounding factors of surgical skill, technique, experience and postoperative care. All surgery will be performed by this surgeon.

All eligible patients who fulfil the selection criteria during the recruiting period will be invited to participate. Inclusion criteria will be all patients aged 18 to 90 who will be undergoing rotator cuff repair, subacromial decompression or excision of the distal clavicle by the surgeon. Exclusion criteria will be: (i) grade IV gleno-humeral arthritis of the shoulder undergoing operation diagnosed on arthroscopy (ii) preoperative shoulder stiffness (iii) concomitant surgical procedure except for rotator cuff repair, subacromial decompression, distal clavicular resection, debridement, bursectomy, synovectomy or biceps tenodesis (iv) superior labral tear from anterior to posterior (SLAP) repair (v) inability to answer questions by phone (vi) poor English language skills.

### Ethics

The Monash University Human Research Ethics Committee has approved the study.

All participants will provide informed written consent witnessed by an external observer.

### Study procedure

#### Interventions

The standard surgical protocol for rotator cuff repair, subacromial decompression and distal clavicular resection will be followed. Participants will be asked to cease all non-steroidal antiinflammatory drugs two weeks prior to commencement of the study. All operations will be performed under general anaesthesia using a standardised method consisting of propofol for induction and sevoflurane with air to maintain heart rate, blood pressure and respiratory rate within normal limits and spontaneous ventilation on a laryngeal mask unless contraindicated.

As part of standard management, intraoperatively patients will receive metoclopramide 10 mg intravenously for nausea as well as a morphine 10 mg, fentanyl 100mcg, paracetamol 1 g and parecoxib 40 mg intravenously for pain. Postoperatively patients will receive oral slow release paracetamol 665 mg *x*2 every 8 h, slow release oxycodone 10 mg every 12 h and have quick release oxycodone 5 mg available for break through pain as required. This management, including dosages and medications, may be adjusted to accommodate for individual patient factors.

At the beginning of surgery, prior to the insertion of the arthroscope a bolus dose of 20mls of 0.75 % ropivacaine will also be injected into the glenohumeral joint, subacromial space and/or acromioclavicular joint as required. At the completion of the operation 20mls of 1.0 % ropivacaine will be injected in to the same space(s).

#### Outcome assessment

Baseline data collected from participant will include sex, age, dominant arm, shoulder side undergoing operation, history of diabetes mellitus and present treatment for diabetes mellitus, third party compensation status, history of depression, time since diagnosis of depression, whether participant is presently receiving treatment for depression and whether and type of previous surgery the participant has had on the shoulder undergoing operation (see Additional file 1).

Preoperatively participants will have range of motion assessed and be asked to complete a questionnaire encompassing pain, analgesia, sleep and mood (see Additional file 2). Range of motion of the shoulder will be assessed for each of the following planes of motion: active elevation, passive glenohumeral abduction with stabilisation of the scapula, passive external rotation in adduction and passive internal rotation in 90°, or maximal, abduction. This will be done using a goniometer. Average pain, worst pain at rest whilst seated with the arm at the side, worst pain with movement and worst pain at night over the last 24 h will be assessed using a visual numerical rating scale (NRS). The NRS pain scale is an 11 point scale where 0 = no pain and 10 = worst possible pain. Participants will be asked to detail the types of analgesia, dosage and treatment they have received in the last 24 h. Sleep will be assessed using a visual NRS sleep scale where 0 = worst possible sleep and 10 = best possible sleep. Participants will be asked what type and dosage of mediation (if any) they have taken to help them sleep in the last 24 h. Last, participants will be provided with a Patient Health Questionnaire – 9 (PHQ-9) that will assess their mood and depressive symptoms over the past two weeks and they will be classified as depressed or not depressed according to the DSM-IV criteria for a major depressive episode [35]. The PHQ-9 has a sensitivity of 98 % and specificity of 80 % for major depressive disorder when using the cutoff of 11 points or greater and allows for stratification of depression severity [35, 36].

Postoperatively participants will be clinically followed up at multiple time points. The operating surgeon will assess them at both their first and final postoperative consultations. The first consultation around the time of suture removal, and the second consultation three months after the operation. In addition the investigator will contact them by phone postoperatively on days two, five and seven and additionally at weeks four, seven and ten. At both postoperative surgeon clinical assessments the participants will asked to complete the PHQ-9. This will assess their mood over the previous two weeks. At their final postoperative consultation the range of motion of their treated shoulder will also be assessed. The senior surgeon will determine clinically whether or not the patient has ‘postoperative stiff shoulder’.

During each of the follow up phone calls, participants will be asked to complete the same pain questionnaires as preoperatively with the use of a verbal NRS and to detail the types of analgesia, dosage and any other treatment they have received in the last 24 h. The preoperative sleep assessment, using a verbal NRS, will be preformed postoperatively at these times. Participants will be asked what type and dosage of mediation (if any) they have taken to help them sleep in the last 24 h. Lastly patients will be asked to describe their mood over the past 24 h and also describe any events or stressors that may have impacted upon their pain, sleep or mood. This will be recorded on specifically designed form (see Additional file 3). Therefore each participant will have six phone assessments over the three months.

### Sample size

Using the PASS^13^ software a two sample *T*-test assuming equal variance in both groups was performed. The power was set at 0.80 and the significance level (α) at 0.05. A clinically meaningful difference between the postoperative stiff shoulder and non-postoperative stiff shoulder groups was defined as a mean difference of 2 pain units [37, 38] on a scale of 0–10 and the standard deviation of these means was estimated from a previous randomised controlled trial of postoperative shoulder pain to be 2.20 [39]. Assuming a low incidence of postoperative stiff shoulder of 10 % a sample size of 110 patients was determined. To allow for loss to follow up and postoperative exclusion a 20 % margin was included resulting in a required sample size of 132 participants.

### Analysis

The incidence of postoperative stiff shoulder will be determined using the treating surgeon’s clinical diagnosis informed by the Buchbinder criteria of restriction of motion of greater 30° in two or more planes of shoulder motion [40].

Patient demographics, baseline range of motion, pain, sleep and depression scores and outcome data will be presented for both outcome groups (postoperative stiff shoulder and no postoperative stiff shoulder).

The principal analysis will involve assessing whether or not a relationship exists between average pain scores and development of postoperative stiff shoulder. This will be by determining if there is a significant difference of the mean of the “average pain scores over the last 24 h” between both outcome groups at each recorded time point and over predetermined periods (first week postoperatively, first month postoperatively and from week four to ten postoperatively), adjusted for multiple comparisons using the Holm’s step down procedure [41].

Secondary analysis will assess depression and its relationship with PSS. A history of depression diagnosis as well as active depression at each time point, as assessed by the PHQ-9, will be assessed for an association with a diagnosis of PSS adjusted and not adjusted for baseline depression, and adjusted for multiple comparisons using the Holm’s step down procedure [41].

This study will also report the incidences of postoperative stiff shoulder after rotator cuff repair as well as subacromial decompression and excision of the distal clavicle not in association with rotator cuff repair.

Finally a series of range of motion criteria will be used to objectively determine a range of incidences of PSS in the same cohort (Table 1).

**Table 1:** Criteria for diagnosing postoperative stiff shoulder

Criteria for diagnosing postoperative stiff shoulder
Clinician’s diagnosis
Range of motion thresholds in two planes, both required • Active elevation <100 degrees AND • Passive external rotation <30 degrees
Range of motion thresholds in two planes, at least one required • Active elevation <100 degrees OR • Passive external rotation <30 degrees
Restricted range of motion compared to contralateral side, at least two planes • Restriction of >30 degrees in TWO or more planes of movement
Restricted range of motion compared to contralateral side, at least three planes • Restriction of >30 degrees in THREE or more planes of movement

## Discussion

The aims of the study will be to provide greater insight into the incidence of PSS post rotator cuff repair and report incidences of PSS post subacromial decompression and/or excision of the distal clavicle not in association with rotator cuff repair. A further aim is to identify whether increased pain levels can be used as a marker of patients who may benefit from early intervention to prevent or manage PSS. and to assess whether depression is a risk factor for the development of PSS. Finally we aim to compare and contrast the incidences of PSS determined by different objective clinical criteria in the same patient cohort.

The results of this trial we anticipate will contribute to our clinical understanding of the development of the postoperative stiff shoulder therefore improving clinical care and peri-operative information for patients. In addition it will provide a platform for discussion of how to objectively define this condition with the aim of assisting the development of a consensus definition.

Strengths of our prospective study will include the involvement of a single surgeon with set surgical protocols, statistical power to detect a clinically meaningful difference in pain scores, and the ability to assess a temporal relationship between pain, depression and postoperative stiff shoulder through the use of a cohort study. In addition the study will have the ability to determine the incidence rate of PSS after rotator cuff repair as well as in patients having a subacromial decompression without rotator cuff repair and also those undergoing an excision of the distal clavicle not in association with rotator cuff repair. These figures are absent from the current literature.

## Additional files

Additional file 1:
**Participant Questionnaire – Demographics and History of Depression.** Preoperative demographics and history of depression questionnaire. (DOCX 69 kb) Additional file 2:
**Participant Questionnaire – Preoperative Pain, Sleep and Analgesia.** Preoperative pain, sleep and analgesia questionnaire. (DOCX 88 kb) Additional file 3:
**Participant Questionnaire – Postoperative Pain, Sleep and Analgesia.** Postoperative Pain, Sleep and Analgesia. (DOCX 102 kb)

## Abbreviations

NRS: numerical rating scale
PHQ-9: patient health questionnaire – 9
PSS: postoperative stiff shoulder
SD: standard deviation
SLAP: superior labral tear from anterior to posterior
