# Supplementary material for: Pain, depression and the postoperative stiff shoulder
Source: BMC Musculoskelet Disord. 2015 Dec 4;16:376. doi: 10.1186/s12891-015-0841-6 (PMC4669665; doi:10.1186/s12891-015-0841-6)
Supplement: Additional file 1: — Participant Questionnaire – Demographics and History of Depression. Preoperative demographics and history of depression questionnaire. (DOCX 69 kb) [file 12891_2015_841_MOESM1_ESM.docx]

**Participant Questionnaire – Demographics and History of Depression**

Date: ________

Participant code: ________ Day/Week: PREOPERATIVE

1. What is your sex? (Please tick relevant response)

**☐** Female

**☐** Male

2. What is your age in years? (Please write on line below)

________ years

3. What is your preferred contact phone number? (The researchers will use this number to contact you to conduct follow up questionnaires)

Preferred __________________________

Other __________________________

4. What shoulder(s) of yours is to undergo an operation? (Please tick all relevant responses)

**☐** Left

**☐** Right

5. Is this your dominant arm?

☐ Yes

☐ No

6. Have you ever been diagnosed with diabetes mellitus, type 1 or 2? (Please tick relevant response and if unsure please ask researcher)

☐ Type 1

☐ Type 2

☐ No

***Please turn over***

7. If you have been diagnosed with diabetes mellitus type 1 or 2 how are you managing your diabetes? (Please tick relevant response)

☐ Diet and lifestyle only

☐ Oral hypoglycaemic agents (metformin, gliclazide)

☐ Insulin injections

☐ Not applicable

8. Are you currently involved in a third party compensation claim related to your shoulder that is to undergo an operation? (Please tick relevant response)

☐ Yes

☐ No

9. Have you ever been diagnosed by a medical doctor (general practitioner or psychiatrist) with depression? (Please tick relevant response)

☐ Yes

☐ No

10. If you answered yes to question 9, when was this diagnosis first made? (Please tick relevant response)

☐ In the last 12 months

☐ 12 months to 5 years ago

☐ Longer than 5 years ago

☐ Not applicable

11. If you answered yes to question 9, are you currently receiving treatment for your depression? This may include medication, psychotherapy with a psychiatrist or psychologist as well as other therapies such as electroconvulsive therapy. (Please tick relevant response)

**☐** Yes

**☐** No

☐ Not applicable

12. Have you ever undergone any surgery on the shoulder that you are to have an operation on? If yes, please describe what operation and when below

**☐** Yes **☐** No

______________________________________________________________________________________________________________________________________________________
