# Supplementary material for: Pain, depression and the postoperative stiff shoulder
Source: BMC Musculoskelet Disord. 2015 Dec 4;16:376. doi: 10.1186/s12891-015-0841-6 (PMC4669665; doi:10.1186/s12891-015-0841-6)
Supplement: Additional file 3: — Participant Questionnaire – Postoperative Pain, Sleep and Analgesia. Postoperative Pain, Sleep and Analgesia. (DOCX 102 kb) [file 12891_2015_841_MOESM3_ESM.docx]

**Participant Questionnaire – Postoperative Pain, Sleep and Analgesia**

Date: ________ Days/weeks post op: ___________________________

Participant code: ________

1. On a scale 0 to 10, 0 being no pain and 10 being the worst possible pain what number best describes your **average level of shoulder pain** over the **last 24 hours.**

| **NO PAIN** | | |  | | | | **MODERATE PAIN** | | |  | | | | **WORST POSSIBLE PAIN** | |
| --- | --- | --- | --- | --- | --- | --- | --- | --- | --- | --- | --- | --- | --- | --- | --- |
| **0** | **1** | | **2** | **3** | **4** | | **5** | **6** | | **7** | **8** | **9** | | **10** |  |

_________________________________________________________________________________________________________

2. On a scale 0 to 10, 0 being no pain and 10 being the worst possible pain what number best describes your **worst shoulder pain at rest** **whilst seated** over the **last 24 hours.**

| **NO PAIN** | | |  | | | | **MODERATE PAIN** | | |  | | | | **WORST POSSIBLE PAIN** | |
| --- | --- | --- | --- | --- | --- | --- | --- | --- | --- | --- | --- | --- | --- | --- | --- |
| **0** | **1** | | **2** | **3** | **4** | | **5** | **6** | | **7** | **8** | **9** | | **10** |  |

_________________________________________________________________________________________________________

3. On a scale 0 to 10, 0 being no pain and 10 being the worst possible pain what number best describes your **worst shoulder pain with movement** over the **last 24 hours.**

| **NO PAIN** | | |  | | | | **MODERATE PAIN** | | |  | | | | **WORST POSSIBLE PAIN** | |
| --- | --- | --- | --- | --- | --- | --- | --- | --- | --- | --- | --- | --- | --- | --- | --- |
| **0** | **1** | | **2** | **3** | **4** | | **5** | **6** | | **7** | **8** | **9** | | **10** |  |

_________________________________________________________________________________________________________

4. On a scale 0 to 10, 0 being no pain and 10 being the worst possible pain what number best describes your **worst shoulder pain at night** over the **last 24 hours.**

| **NO PAIN** | | |  | | | | **MODERATE PAIN** | | |  | | | | **WORST POSSIBLE PAIN** | |
| --- | --- | --- | --- | --- | --- | --- | --- | --- | --- | --- | --- | --- | --- | --- | --- |
| **0** | **1** | | **2** | **3** | **4** | | **5** | **6** | | **7** | **8** | **9** | | **10** |  |

5. What types of pain medication and treatment have you had over the last 24 hours? (Please tick)

**☐** Paracetamol (Panadol, Panadol Osteo)

**☐** Codeine (Panadeine, Panadeine Extra, Panadeine Forte)

**☐** Non steroidal anti inflammatory drugs (Ibuprofen, diclofenac, Nurofen, Voltaren)

**☐** Tramadol

**☐** Opioids (Morphine, OxyContin, Endone)

**☐** Tri-cyclic antidepressants (Amitriptyline, Endep)

**☐** Pregabalin, gabapentin (Lyrica, Neurontin)

**☐** Massage

**☐** Acupuncture

**☐** Heat or ice packs

**☐** Exercise program

**☐** Other (Please describe below)

____________________________________________

____________________________________________

***Please turn over***

6. How many and what dosage of pain medication did you take? E.g. Panadol (500mgs) 2 tablets 3 times a day.

___________________________________________________________________________________________________________________________________________________________________________________________________

7. On a scale of 0 to 10, 0 being worst possible sleep and 10 being the best possible sleep how did you sleep last night?

| **WORST POSSIBLE SLEEP** | | |  | | | | **AVERAGE SLEEP** | | |  | | | | **BEST POSSIBLE SLEEP** | |
| --- | --- | --- | --- | --- | --- | --- | --- | --- | --- | --- | --- | --- | --- | --- | --- |
| **0** | **1** | | **2** | **3** | **4** | | **5** | **6** | | **7** | **8** | **9** | | **10** |  |

8. Have you taken any medication to help you sleep in the past 24 hours?

**☐** Yes

**☐** No

9. (If participant has answered yes to question 8) Please outline what medication you have taken in the past 24 hours to help you sleep?

_____________________________________________________________________________________________________________________________________________________________________________________________________________________________________________________________________________________________

10. Could you please describe your mood over the past 24 hours?

___________________________________________________________________________________________________________________________________________________________________________________________________

11. Lastly, has anything out of the ordinary occurred which may have impacted upon your pain, sleep or mood? If so please describe

______________________________________________________________________________________________________________________________________________________________________________________________________________________________________________________________________________________________________________________________________________________________________________________________
